# Revisiting the Diversity of Nasolabial Flaps in Reconstructive Surgery

**DOI:** 10.7759/cureus.99582

**Published:** 2025-12-18

**Authors:** Ashish S Singhal, Sindhu M

**Affiliations:** 1 Plastic Surgery, Geetanjali Medical College, Udaipur, IND; 2 General Surgery, Kasturba Medical College, Mangalore, Mangalore, IND

**Keywords:** alveolar defect, glabellar defect, nasolabial flap, palatal defect, tongue defect

## Abstract

Background: The nasolabial flap is a regional flap with vascularity based on the facial artery inferiorly and the angular artery superiorly. Depending on the area to be covered, it can be islanded or pedicled and performed in a single-stage or two-stage procedure. The tissue laxity in this area provides a wider arc of rotation, and the good color match adds to the other advantages of this flap. Generally, it is used to cover nasal, lip, and alveolar defects. It is especially useful in patients with multiple comorbidities, in whom major flaps cannot be planned.

Methodology: This is a retrospective descriptive study of 19 patients who underwent closure of various facial and intraoral defects with nasolabial flaps and were admitted to a tertiary care hospital between January 2019 and March 2024. The various parameters analyzed were the age, gender, comorbidities, size of defect, duration of hospital stay, wound dehiscence, flap necrosis, and the type of nasolabial flap that was used.

Results: In patients in whom the facial defects were small and when free flaps were not possible due to poor general conditions and comorbidities, nasolabial flaps were used. Wound healing was good with no flap necrosis. Nasolabial flaps were used for covering defects over the dorsum, root, and ala of the nose, tongue, hard palate, buccal mucosa, glabella, upper and lower lip, and angle of the mouth.

Conclusion: Nasolabial flaps are highly robust and useful in patients with comorbidities and for smaller defects. They provide good aesthetic and functional outcomes in all patients.

## Introduction

The nasolabial flap is a very robust and vascular flap. Its vascularity is based on the facial artery inferiorly [1] and the angular artery superiorly [2]. Depending on the area to be covered, the procedure can be performed in a single-stage or a two-stage procedure [3]. Frequently, it is used for covering nasal, lip, and alveolar defects. It is especially useful in patients with multiple comorbidities, in whom major flaps cannot be planned.

Depending on the manner of transfer of the flap, it can be advanced, transpositioned, tunneled, or interpolated. In patients with multiple comorbidities, where every trip to the operating room is an anesthetic challenge, single-stage procedures are useful (without a secondary surgery for flap division). In addition, the donor site is closed primarily without another skin graft.

There appears to be a paucity of studies, particularly on the nasolabial flap, highlighting its ability to address most facial defects in and around the nose and mouth with satisfactory outcomes [4]. This is particularly important in low-income or developing countries, where multiple comorbidities and patients' poor general condition necessitate the use of single-stage local flaps.

This retrospective study demonstrates the wide range of facial defects that nasolabial flaps have successfully addressed. Although multiple case reports have been published on the closure of defects involving different facial regions using various flaps, including nasolabial flaps, few studies are available in the Indian setting on the use and success of nasolabial flaps. Hence, studies on this flap can facilitate greater use in the future due to its ease, shorter surgical duration, and good aesthetic outcomes. Our study primarily focuses on the nasolabial flap utilized to address defects of the tongue, palate, buccal mucosa, glabella, nose, and lip.

## Materials and methods

We retrospectively analyzed 19 patients from January 2019 to March 2024 who had undergone flap covers using the nasolabial flaps at a tertiary care center for various facial defects related to trauma or for the excision of orofacial malignant lesions. Institutional ethics committee clearance was obtained prior to the study. All patients aged above 18 years, with or without comorbidities, for whom major flaps could not be planned, were included. Patients who had previously undergone head and neck radiation therapy were excluded.

The study examined patient factors (comorbidities), postoperative outcomes (wound dehiscence, duration of hospital stay, flap necrosis), and, on a follow-up basis, the impact of radiation therapy following nasolabial flap surgery.

Surgical technique

The nasolabial flap was marked out, starting approximately 4 cm from the medial canthus superiorly to about 2 cm from the angle of the mouth inferiorly. Skin and subcutaneous tissue were dissected. The angular artery was specifically preserved for a flap with a superior base. Facial arteries served as the basis for the inferiorly based flap. The flaps were generally sutured to the defect using Vicryl 3-0 interrupted sutures. The defect in the face was closed primarily with interrupted sutures. Postoperatively, the color and vascularity were monitored with a pinprick test for 48-72 hours. The type (intraoral, inferior, or superior) of nasolabial flap was determined based on the site and size of the defect; the maximum flap size was approximately 6 x 3 cm.

In cases of intraoral defects, de-epithelialization of a proximal 1-2 cm was performed to facilitate tunneling. The flap was islanded and pedicled specifically to address tongue defects. The facial artery and vein were essentially kept intact during the dissection of soft tissue to facilitate vascularity and venous drainage. The inferiorly based flaps were used for the lower lip, buccal mucosa, alveolar defects, defects in the floor of the mouth, and tongue. All such patients usually underwent a single-stage procedure. In one case of severe trismus, a coronoidectomy was performed before raising an inferiorly based flap, which was then tunneled to cover the defect in the buccal mucosa. Superiorly based flaps were used for defects of the palate, nose, glabella, and upper lip. In case of a columellar defect, a two-stage superiorly based flap was used following excision of a basal cell carcinoma (BCC). The flap was rotated and placed over the defect, with the donor site sutured primarily. Flap division was done after four weeks.

## Results

Among the 19 patients analyzed in this study, 15 were male, and the mean age was 54.6±15 years. A superiorly based flap was used for seven patients, while an inferiorly based flap was used in 12 patients. The average defect size was approximately 3.5±1.3 x 2.7±1.1 cm. The largest defect size was 6x4 cm, following excision of a leukoplakic patch in the angle of the mouth, and the smallest defect size was 0.5x0.5 cm for a columellar defect following excision of a BCC lesion. The indication for flap placement in the majority of cases was orofacial malignancy. There were three trauma-related defects, where one patient underwent a flap for releasing severe trismus. The mean duration of hospital stay was 6.3±1.1 days. Details are presented in Table 1.

**Table 1:** Patient details, defect characteristics, and the postoperative follow-up of the 19 subjects. Abbreviations: IB - inferiorly based; SB - superiorly based; AF - atrial fibrillation; T2DM - type 2 diabetes mellitus; HTN - hypertension; IHD - ischemic heart disease; CKD - chronic kidney disease; COPD - chronic obstructive pulmonary disease; Ca - carcinoma; SCC - squamous cell carcinoma; BCC - basal cell carcinoma

Flap Used	Age (yrs)	Gender	Diagnosis	Defect Size (cm)	Hospital Stay (Days)	Comorbidities
IB	75	M	SCC lower lip (left side)	4.5x4	8	Type 2 DM, HTN, CKD, COPD, AF
	51	F	SCC lower lip	5x4	7	HTN
	41	F	SCC left buccal mucosa	3x3	7	None
	24	M	Necrosed submental flap lower lip	4x2	8	None
	36	M	Multiple facial lacerations	5x2	7	None
	57	M	Leukoplakia of angle of mouth (right side)	6x4	7	HTN
	70	F	Ca lower lip (right side)	3x2	6	None
	50	M	Ca floor of the mouth	4x2	8	None
	49	M	Severe trismus	3x1	6	Hypothyroidism
	74	M	Ca lateral border of tongue	3x2	5	HTN
	63	M	Ca buccal mucosa	3x4	6	IHD, type 2 DM
	75	M	Ca buccal mucosa	4x4	7	IHD, COPD, Type 2 DM
SB	58	M	Ca hard palate	3x2	5	None
	55	M	Defect in left ala nasi	2x2	6	None
	78	M	SCC forehead	3x4	5	HTN
	39	M	Human bite left side of nose	1.5x2	5	None
	48	F	BCC dorsum of nose	4.5x3	6	None
	49	M	BCC columella	0.5x0.5	5	None
	45	M	Ca hard palate	4x3.5	6	None
Mean	54.6±15			3.5±1.3 x 2.7±1.1	6.3±1.1	

Based on the pinprick test, there were no (n=0) flap-related complications, such as ischemia or venous congestion, in the early postoperative phase. There were no (n=0) instances of flap dehiscence or necrosis. In all 19 cases, the color and contour matches were excellent, resulting in a good aesthetic outcome. In every instance, speech, chewing, and mouth opening were satisfactory. None of the patients (n=0) included in the study had postoperative flap contracture. Though this is a frequently observed complication when islanded pedicled flaps are employed, this complication was not observed since the pedicle with sufficient length (i.e., adequate length, caliber, and orientation to ensure appropriate flap setting and placement) was used with intact artery supply and venous drainage.

Complete coverage of the defect was seen with no incidence of postoperative wound dehiscence. The donor sites were closed primarily, and no donor site complications in the form of wound dehiscence and wound gaping were seen.

On analyzing the comorbidities of the subjects, ischemic complications were expected in patients (n=8) with vascular comorbidities, especially ischemic heart disease and hypertension, due to the underlying atherosclerosis changes in the vessels. However, there was no difference in postoperative outcomes compared with patients without these comorbidities (n=11). An increased duration of wound healing was expected in diabetic patients (n=3). However, no such observations were made, and the duration of healing was grossly the same in all 19 patients. Out of 19 patients, six underwent radiation therapy post-surgery, and out of those, only one patient had epithelial loss, but that was also resolved with daily dressings.

**Figure 1:**
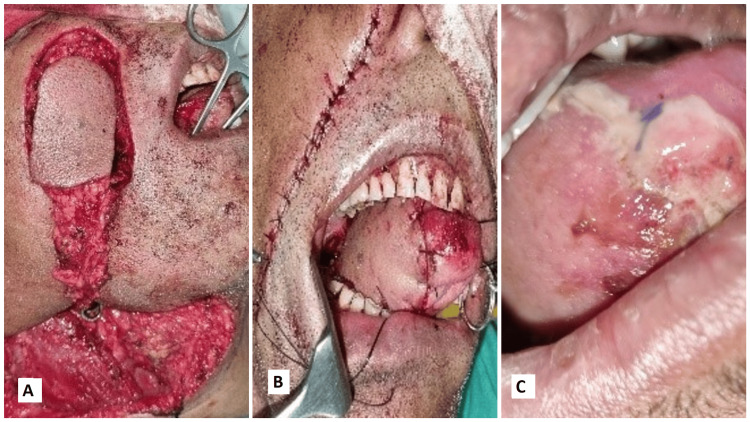
A 74-year-old male patient with a Ca lateral border of the tongue. (A) The use of an inferiorly based islanded nasolabial flap, (B) a B-flap inset over the tongue defect, (C) postoperative follow-up of a well-settled flap. Ca - carcinoma

**Figure 2:**
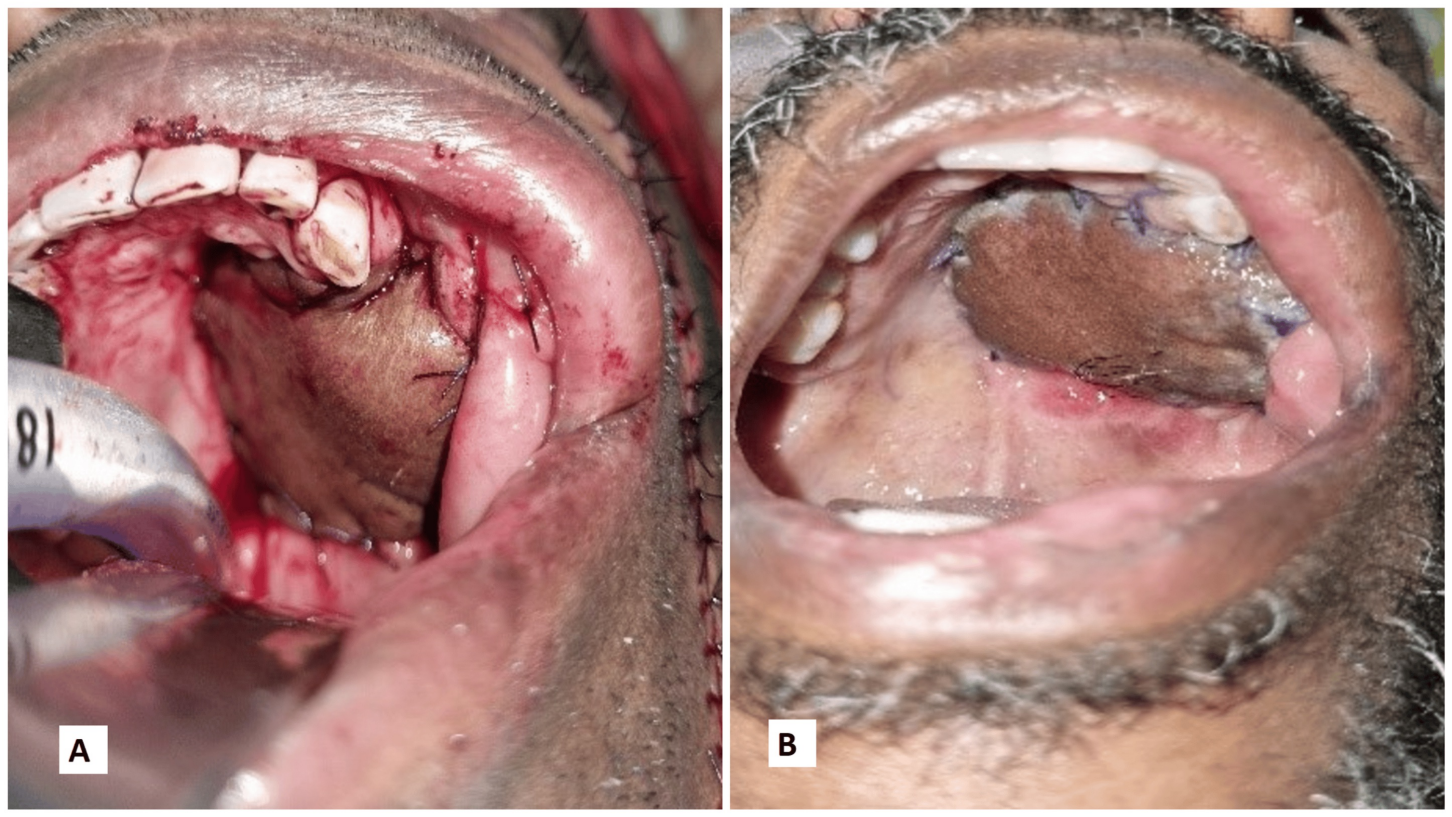
A 45-year-old male patient with Ca hard palate. (A) Intraoperative superiorly based flap inset on palate, (B) postop follow-up showing well-settled flap. Ca - carcinoma

**Figure 3:**
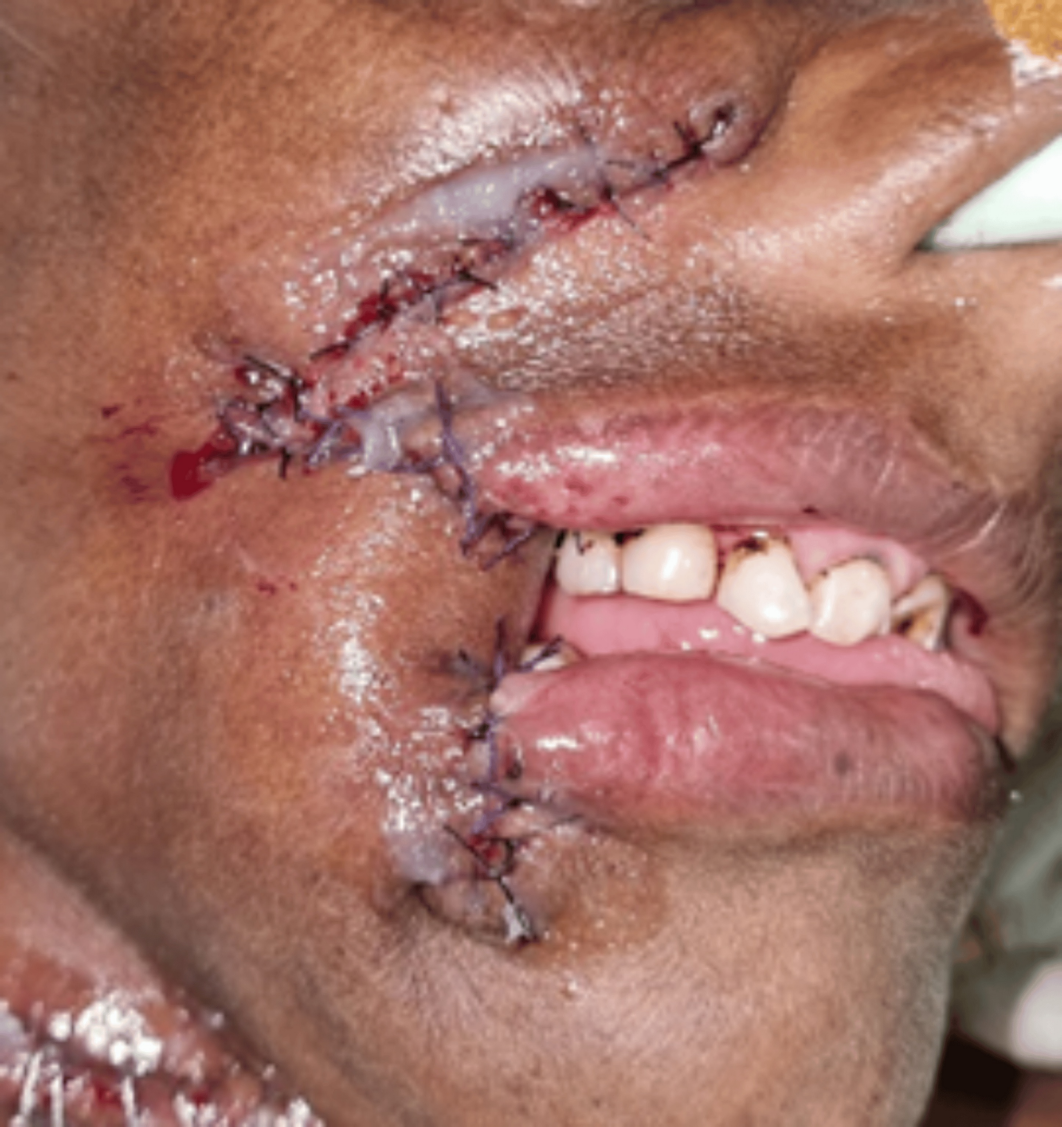
A 75-year-old male patient with a Ca right buccal mucosa defect covered with an inferiorly based nasolabial flap. Ca - carcinoma

**Figure 4:**
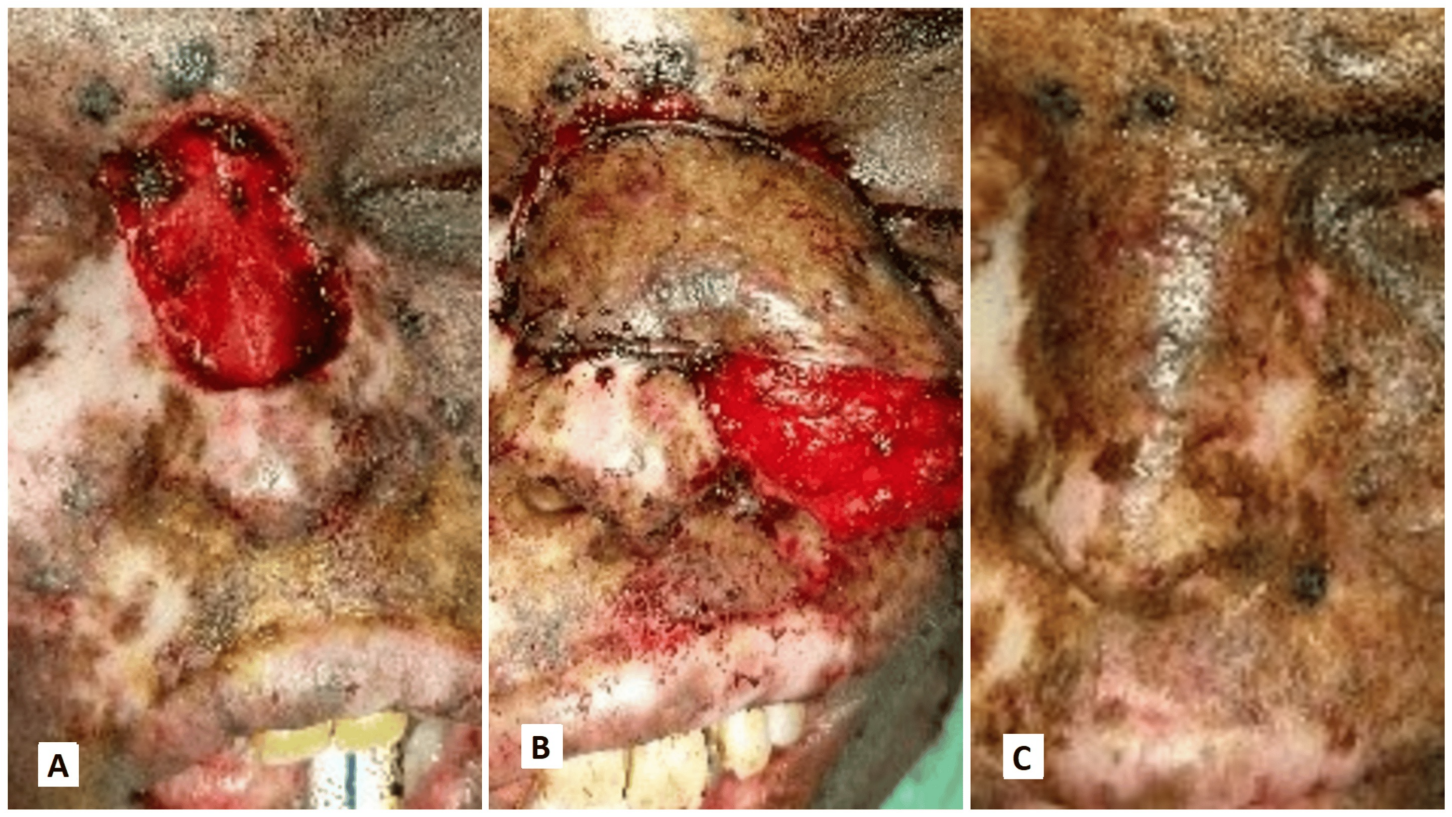
A 48-year-old female patient with BCC on the dorsum of the nose. (A) Defect on the dorsum of the nose post-excision, (B) superiorly based nasolabial flap cover intraoperatively, (C) follow-up with good contour of the nose. BCC - basal cell carcinoma

**Figure 5:**
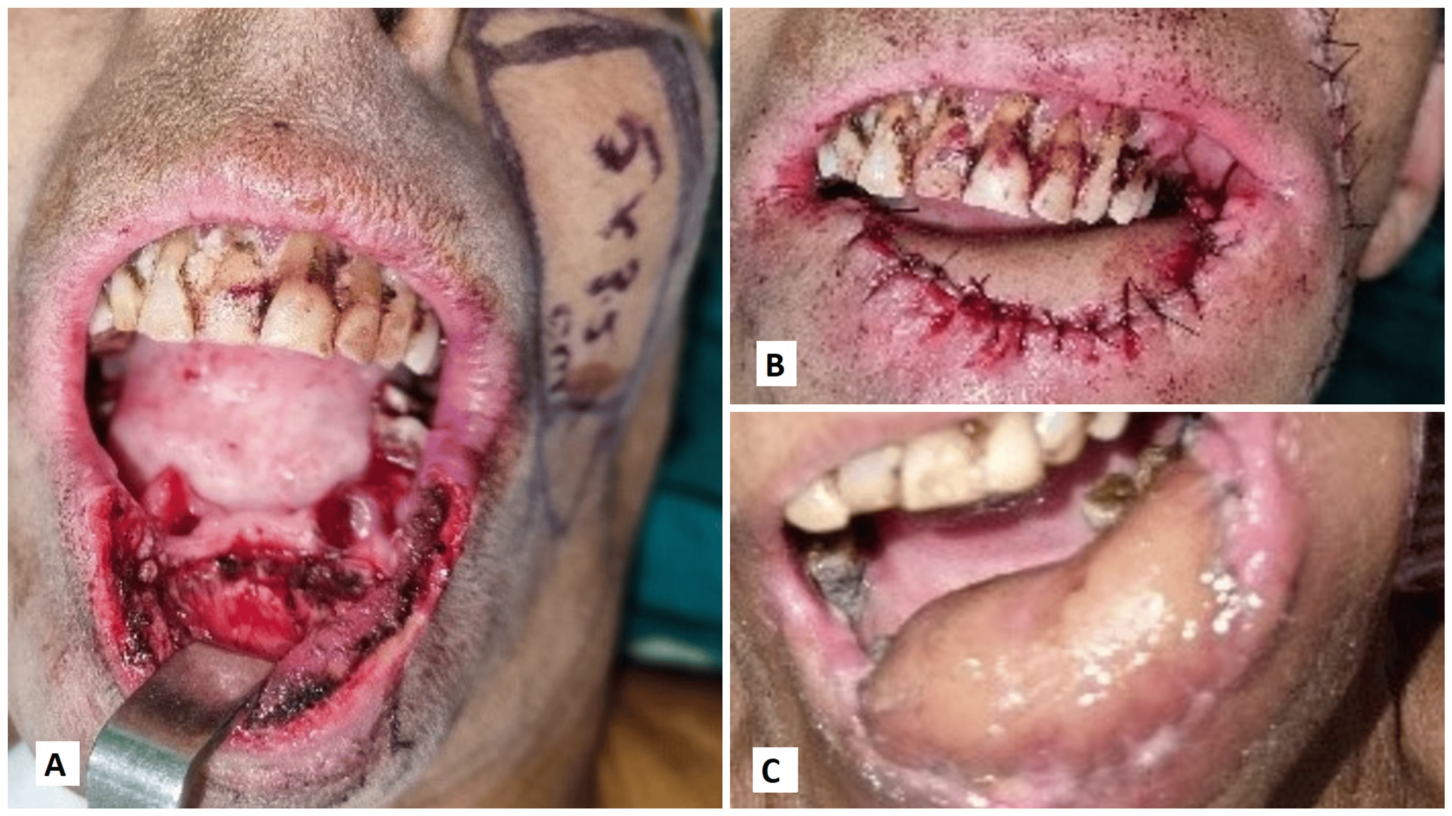
A 51-year-old female patient with Ca lower lip. (A) Lower lip and alveolar defect post-excision, (B) defect covered using inferiorly based nasolabial flaps, (C) well-settled flap on follow-up. Ca - carcinoma

**Figure 6:**
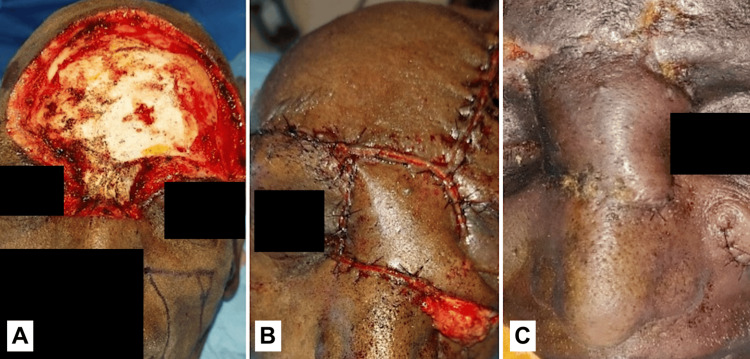
A 78-year-old male patient with SCC of the forehead. (A) Defect of forehead, (B) cover of a glabellar defect with a superiorly based nasolabial flap, and forehead with rotation flap, (C) postop well-settled nasolabial flap. SCC - squamous cell carcinoma

The following images present the results of nasolabial flaps for various defects. Figure 1 shows the use of an inferiorly based islanded nasolabial flap to cover a tongue defect with good postoperative tongue movements and satisfactory speech and swallowing. Figure 2 shows the reconstruction of the hard palate with a superiorly based nasolabial flap. Figure 3 shows the covering of a right buccal mucosal defect with an inferiorly based nasolabial flap. Figure 4 shows a superiorly based nasolabial flap cover for a dorsum of nose defect with satisfactory postoperative color match and contour. Figure 5 shows the lower lip and alveolar defect covered using inferiorly based nasolabial flaps. Figure 6 shows the closure of a glabellar defect with a superiorly based nasolabial flap and a forehead defect with a rotation flap.

## Discussion

Nasolabial flaps are described in various orofacial defects [4]. Tongue defects corrected with nasolabial flaps may not provide the entire function of the normal tongue. Nueangkhota et al. conducted a study utilizing contralateral nasolabial flaps for the reconstruction of tongue abnormalities, with successful outcomes [5]. The functional outcome following flap uptake was good. The rationale for employing contralateral flaps was that the likelihood of malignancy recurrence in contralateral tissues was lower than in ipsilateral tissues. A significant drawback of employing a contralateral flap is the necessity for an extended pedicle to avert postoperative contracture, thereby elevating the risk of damage to the facial artery. In the present study, the closure of tongue defects was performed using ipsilateral flaps; no instances of malignancy recurrence or flap contracture were observed.

Nasolabial and forehead flaps have been employed for columellar reconstruction. Our study included a staged superiorly based nasolabial flap. Nasal obstruction, resulting from columellar collapse due to insufficient cartilage, has been documented after columellar restoration [6]. However, in this study, we did not observe this consequence. Dolan et al. utilized an island pedicled musculocutaneous nasolabial flap for the repair of a total columellar defect [7]. In our study, a flap based on the angular artery was utilized for the reconstruction of the columella after the excision of a BCC lesion. The flap division performed four weeks later demonstrated satisfactory flap uptake with no signs of necrosis.

For coverage of buccal mucosal defects, various flaps, such as tongue, temporalis, and radial forearm free flaps, have been conventionally employed; nasolabial flaps have demonstrated encouraging outcomes for this purpose. Lazaridis et al. did a study on the coverage of buccal mucosal abnormalities using nasolabial flaps [8]. It was determined that the buccal region's rich vascularization, supplied by the infraorbital, buccal, masseteric, and transverse facial arteries, enabled the successful use of nasolabial flaps even when the facial artery was ligated during neck dissection. Our study employed inferiorly based nasolabial flaps to cover buccal mucosal abnormalities, conducted as a single-stage procedure. It was ensured that the facial artery was not damaged and remained intact throughout the neck dissection.

The application of nasolabial flaps for the reconstruction of glabellar defects also remains under-researched. Iida et al. utilized bilateral retroangular flaps for the reconstruction of a glabellar defect [9]. The deformity resulting from the excision of a hemangioma on the glabella extended to the tip of the nose. Due to the substantial extent of the defect, bilateral flaps were necessitated, utilizing retrograde flow from the angular artery. Our study employed a superiorly based nasolabial flap for the rebuilding of a defect on the glabella subsequent to the excision of a squamous cell carcinoma lesion. The glabella, as a prominent facial area, necessitates an optimal color and contour match; therefore, employing a nasolabial flap is particularly advantageous in this context. Carcinomatous lesions pose a larger challenge due to the necessity of a wider margin to prevent recurrence. The favorable results of our study indicate that nasolabial flaps for glabellar defect reconstruction can be employed more extensively.

Reconstruction of the nose is an aesthetically challenging procedure. Traditionally, nasal reconstruction adheres to the subunit concept; if more than 50% of a subunit is affected, complete excision of the subunit is recommended, followed by reconstruction. This is to guarantee the preservation of symmetry and face contour. The various flaps utilized are a frontal flap, bilobed flap, dorsal nasal flap, and nasolabial flap [10]. Our study employed a superiorly based nasolabial flap for the reconstruction of the dorsum of the nose. The functional and aesthetic outcomes were satisfactory, with good contour and color match.

Tan et al. investigated the application of nasolabial flaps for the reconstruction of lip deformities [11]. The Webster flap and Karapandzic flap are employed for lip defect reconstruction; nevertheless, they are associated with the drawbacks of microstomia and diminished lip function [12]. The Webster flap distorts the modiolus, hence interfering with muscle function. The utilization of the nasolabial flap proved advantageous for lip deformities involving the commissure. No instances of postoperative salivary drooling were documented. The facial symmetry and aesthetic result were favorable. The functional outcome in the form of mouth opening was satisfactory.

The use of nasolabial flaps for the coverage of palatal defects has not been extensively addressed. Mohan et al. have detailed the application of the tongue flap for palatal reconstruction [13]. The utilization of the buccal pad of fat for the closure of palatal abnormalities has been studied [14]. A drawback of employing the tongue flap is the potential compromise of the tongue's functional capacity. The present study demonstrates the successful application of the nasolabial flap for the coverage of palate defects, achieving favorable functional and aesthetic results.

Radiotherapy after flap reconstruction has been shown to induce changes, including the pin-cushioning effect [15]. Post-surgical radiotherapy has been demonstrated to induce trismus and mucositis, which can adversely affect quality of life and flap viability [16]. Analysis of radiation therapy follow-up after nasolabial flap surgery was done in the study. Among the 19 patients, six received radiation therapy after nasolabial flap surgery. They were monitored and assessed to study the effects of radiation therapy on flap uptake. Follow-up with five patients exhibited favorable outcomes after radiation therapy subsequent to nasolabial flap reconstruction. No adverse effects, such as trismus, mucositis, or flap necrosis, were observed. One patient exhibited a minor region of epithelial loss, which resolved with daily dressings.

Although the wide range of indications included in the study is a strength that highlights the versatile applicability of the nasolabial flap, it also introduces certain limitations. The varied nature of defect sites, tissue requirements, and flap modifications limits the ability to draw equivocal comparisons. Additionally, the study represents the experience of a single center and operating surgeon; the broader applicability of the study findings may be restricted.

## Conclusions

The present study demonstrates the ease, reliability, and efficacy of successful outcomes for multiple orofacial defects, such as nasal, lip, buccal mucosa, glabella, palate, and tongue reconstruction, using the nasolabial flap. The nasolabial flap can be more widely applied for palatal and glabellar defects. Nasolabial flaps for covering multiple types of facial defects, as a routine practice, can be strongly considered for small- to medium-sized defects and even in patients unfit for long-duration surgeries, such as free flaps.
